# Acute Lumbar Schmorl’s Node Following Chiropractic Adjustment

**DOI:** 10.7759/cureus.25209

**Published:** 2022-05-22

**Authors:** Stephen Albano, Mary Grace Bacani, Anthony Alastra

**Affiliations:** 1 Neurosurgery, Desert Regional Medical Center, Palm Springs, USA; 2 Intensive Care Unit, Desert Regional Medical Center, Palm Springs, USA

**Keywords:** pain management, spine, low back pain, chiropractor, herniated disc, schmorl’s node

## Abstract

Schmorl’s nodes are typically an incidental finding whereby a disc herniates into the vertebral body. The clinical course is rarely symptomatic. Here, we present a 41-year-old male who, following chiropractic manipulation, developed an acute Schmorl’s node. The patient was managed successfully with conservative measures. A 41-year-old male with acute on chronic low back pain following a chiropractic adjustment. Imaging demonstrated the development of an acute Schmorl’s node of the lumbar four-five disc into the lumbar 4 vertebral body after a chiropractic adjustment. He was treated with conservative measures with over 85% relief of back pain. Acute Schmorl’s nodes can develop after the chiropractic adjustment. In the case presented, conservative measures resulted in 85% improvement in pain.

## Introduction

Schmorl’s nodes are intervertebral discs that have herniated into the vertebral body. Schmorl’s nodes are asymptomatic and typically an incidental finding with an incidence as high as 76% and a slightly higher predominance in males [1,2]. They are most commonly located in the thoracolumbar spine [1,3-5]. It is possible to see the bony deformity on a CT scan, but due to its asymptomatic nature, MRI is not typically performed. In a minority of patients, Schmorl’s nodes can be a pain generator, in which case MRI can be of value for assessing options in treatment. Here, we present a patient with an acute exacerbation of low back pain due to the development of a Schmorl’s node following a chiropractic adjustment.

## Case presentation

**Figure 1:**
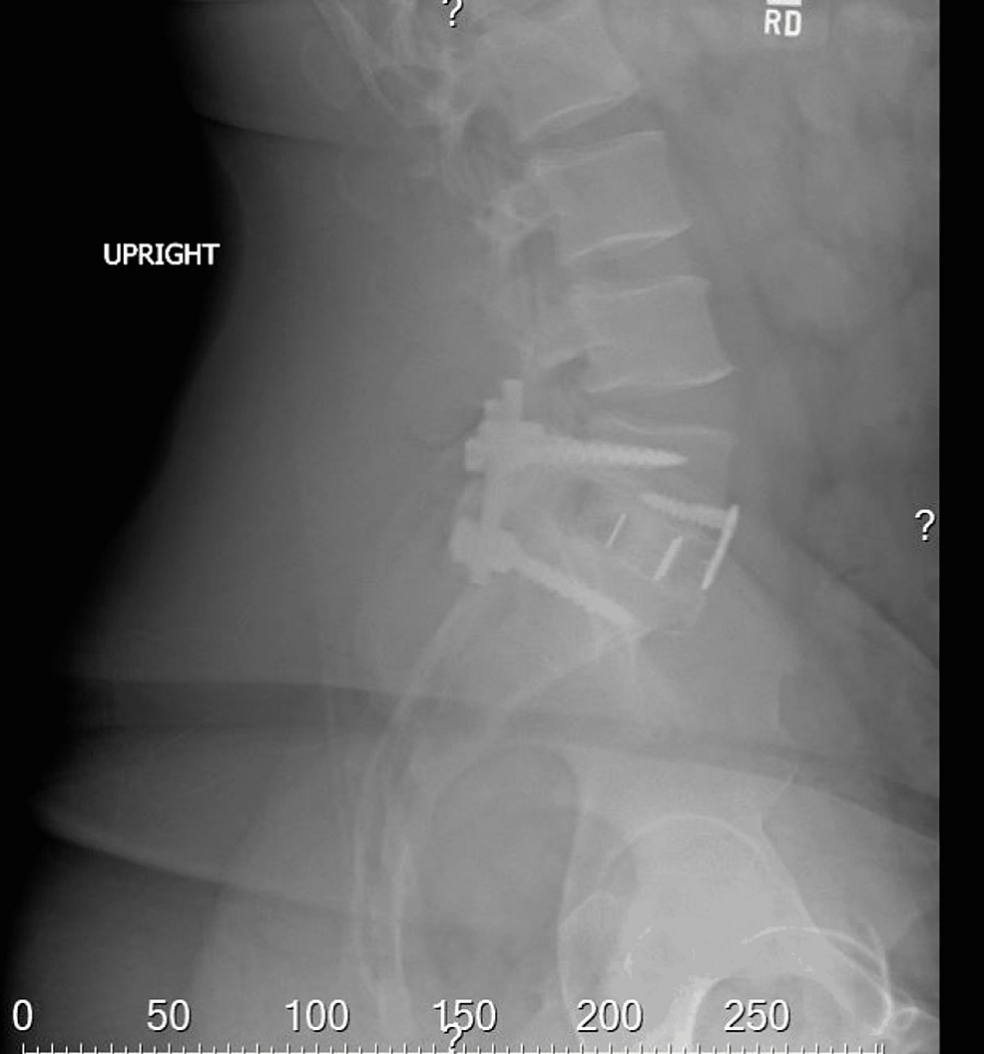
Lateral x-ray of the lumbar spine.

**Figure 2:**
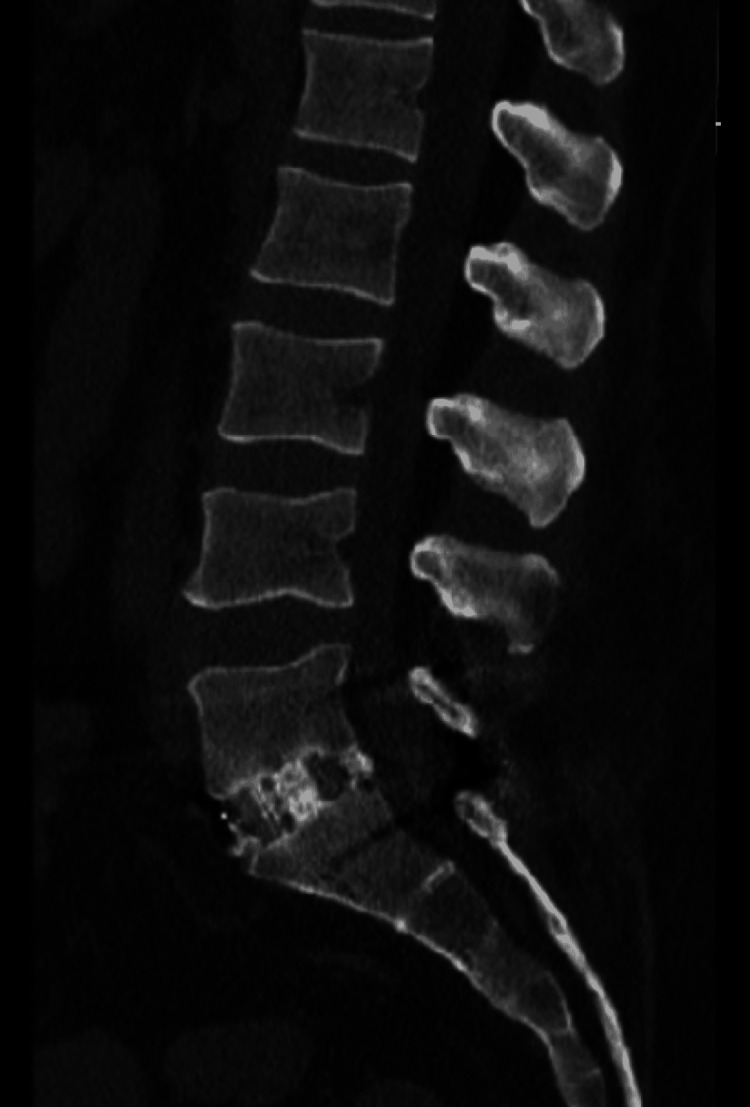
Sagittal CT lumbar spine.

A 41-year-old male with a history of both anterior and posterior L5-S1 instrumented fusions over five years prior presented to the clinic for low back pain. The patient indicated that fourteen weeks prior to the clinic visit, he suffered a mechanical ground-level fall, resulting in low backache. Due to the persistent nature of the pain, his primary care physician ordered an upright lumbar x-ray six weeks later, which did not demonstrate any fractures as shown in Figure 1. Continued persistent pain prompted ordering a non-contrasted CT lumbar spine again, not demonstrating any fractures as shown in Figure 2.

**Figure 3:**
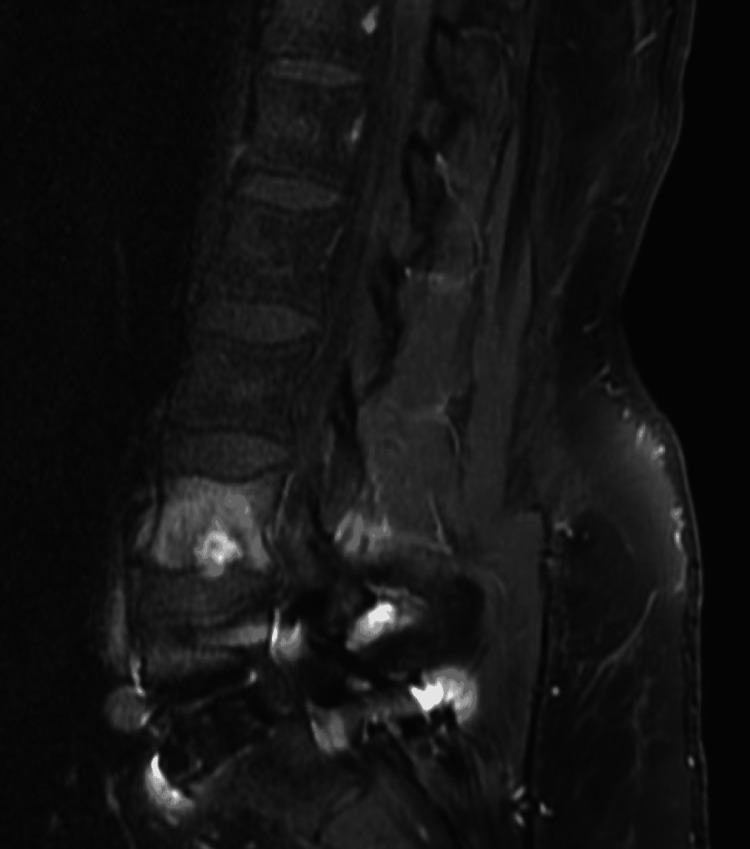
Sagittal MRI short tau inversion recovery sequence with lumbar 4 Schmorl's node.

The patient then went to a chiropractor, who attempted to “crack” his back. Once manipulated, the patient noted an immediate exacerbation of pain with a change in the character of pain. The pain has progressed to involve more than just the midline now, including the right flank and down the posterior right leg. The majority of the pain was described as a muscle spasm in the right flank region. He attempted to sleep the pain away with his current regimen of pain medicines, but this was ineffective and began to affect his ability to ambulate and complete customary activities of daily living (ADL’s), such as changing his own clothes, etc. This prompted ordering a lumbar spine MRI, which is shown in Figure 3. MRI findings prompted a referral to a neurosurgery clinic.

The patient presented to the neurosurgery clinic in a seated walker since upright posture exacerbated the pain. The pain was rated at 5 out of 10 at baseline, with an increase to 10 out of 10 with any movements involving the lower back or upright position. Hydrocodone, carisoprodol, acetaminophen, and pregabalin provided minimal relief, bringing the pain down from 10 out of 10 to 7 out of 10. The patient denied loss of bowel or bladder control and numbness of genitals or rectum. He denied recent fevers, diabetes, immunosuppressive drugs, or intravenous drug use.

On physical exam, the patient did not demonstrate loss of sensation or proprioception. Muscle examination revealed antalgic movements in bilateral lower extremities worse on the left lower extremity, causing right low back pain with exertion. He was non-tender to palpation of the midline lumbar spine or paraspinal muscles bilaterally. There was no erythema or edema of the lower back noted. The body mass index was calculated to be 33.

The patient was prescribed a lumbar support orthotic brace, continued on oral medications with a referral to pain management for consideration of invasive therapies, such as trigger point/epidural steroid injections (ESIs), and then follow-up with physical therapy (PT). Upon follow-up telephone conversations with the patient, he underwent a round of ESI, which after one week significantly improved his symptoms and, after one month with additional gentle PT, alleviated approximately 85% of his symptoms. The patient continues with conservative therapy at this time.

## Discussion

In a minority of patients, Schmorl’s nodes can be a pain generator. A review of the literature reveals suspected mechanisms of action in painful Schmorl’s node development to be primarily axial loading or rotational injuries such as horseback riding, being struck by a motor vehicle, monofin training, and paddling [1,2,4,5]. This is the first reported case of chiropractic manipulation presumed to be the cause as imaging before and after manipulation clearly demonstrates the development of the Schmorl’s node at the lumbar four-five disc space herniating into the lumbar four vertebral body. Despite the literature’s focus on traumatic events as a precursor, the majority of Schmorl’s nodes are non-traumatic and asymptomatic with imaging characteristics of chronic lesions [2,6].

The lack of symptoms may be due to the slow, indolent nature of the presumed etiologies. In one schema, aging degenerates the cartilaginous endplate through which the nucleus pulposus can penetrate if increased intradiscal pressure overcomes the weakened endplate as opposed to the annulus fibrosus [1,7]. Inherent mechanisms within the nucleus pulposus may trigger cytokines and an inflammatory response leading to pain or other symptoms [3]. Other theories for the generation of pain include the fracture of the endplate through which the nucleus pulposus herniates and activates nociceptors in the annulus fibrosus and periosteum of the vertebral bodies that are exacerbated by micromovements, swelling, and inflammation [8,9].

Imaging findings of increased edema within the lumbar four vertebral body support these proposed mechanisms of pain generation from acute Schmorl’s node formation. MRI is the most specific and sensitive for diagnosing Schmorl’s node as a cause of pain [5]. If inflammation and edema are local pain generators, then their presence as increased intensity on T2 and hyperintense short tau inversion recovery (STIR) signal surrounding the acutely formed Schmorl’s node as seen on this patient’s MRI supports that hypothesis in the absence of any other pathology [5-7]. Hyperintense STIR and increased T2 signal are not unique to Schmorl’s nodes; therefore, other pathologies must also be ruled out, such as infection, tumor, or abnormal vasculature.

When the diagnosis of acute Schmorl’s node is reached after appropriate workup, treatment ensues. Treatment is typically conservative, including anti-inflammatories, rest, physical therapy, bracing, or injections, including blocks. Symptoms typically resolve with conservative management, but they can last anywhere from two weeks up to six months [1-3,5,7]. However, the pain can be significant enough to impact ADL’s and impact quality of life as was described in this case. In cases when patients fail conservative management, surgical interventions such as vertebroplasty or discectomy with segmental fusion have been pursued [1,3,7-9].

## Conclusions

Schmorl’s nodes are typically benign incidental findings. However, in certain circumstances, they can be the cause of acute pain and, in rare circumstances, may lead to surgical intervention. The causes of acute Schmorl’s nodes vary, but have been associated with local trauma to that area of the spine. This report is the first documented case of an acute Schmorl’s node following a local traumatic chiropractic adjustment.
